# CMV specific T cell immune response in hepatitis C negative kidney transplant recipients receiving transplant from hepatitis C viremic donors and hepatitis C aviremic donors

**DOI:** 10.1080/0886022X.2022.2072744

**Published:** 2022-05-11

**Authors:** Ambreen Azhar, Makoto Tsujita, Manish Talwar, Vasanthi Balaraman, Anshul Bhalla, James D. Eason, Simonne S. Nouer, Keiichi Sumida, Adam Remport, Isaac E. Hall, Randi Griffin, George Rofaiel, Miklos Z. Molnar

**Affiliations:** a Department of Medicine, Division of Nephrology, Virginia Commonwealth University School of Medicine, Richmond, VA, USA; b James D. Eason Transplant Institute, Methodist University Hospital, Memphis, TN, USA; c Department of Surgery, Division of Transplant Surgery, University of Tennessee Health Science Center, Memphis, TN, USA; d Department of Preventive Medicine, University of Tennessee Health Science Center, Memphis, TN, USA; e Department of Medicine, Division of Nephrology, University of Tennessee Health Science Center, Memphis, TN, USA; f Department of Transplantation and Surgery, Semmelweis University, Budapest, Hungary; g Department of Medicine, Division of Nephrology & Hypertension, University of Utah, Salt Lake City, UT, USA; h Office of Clinical Research, University of Tennessee Health Science Center, Memphis, TN, USA; i Department of Surgery, Division of Transplantation and Advanced Hepatobiliary Surgery, University of Utah, Salt Lake City, UT, USA

**Keywords:** CMV specific T cell immune response, hepatitis C, kidney transplantation

## Abstract

Kidney transplants (KT) from hepatitis C (HCV) viremic donors to HCV negative recipients has shown promising renal outcomes, however, high incidence of cytomegalovirus (CMV) viremia were reported. We performed a prospective cohort study of 52 HCV negative KT recipients from Methodist University Hospital including 41 receiving transplants from HCV aviremic donors and 11 from HCV viremic donors. CMV specific CD4+ and CD8 + T cell immunity was measured by intracellular flow cytometry assay. Primary outcome was the development of positive CMV specific CD4+ and CD8 + T cell immune response in the entire cohort and each subgroup. The association between donor HCV status and CMV specific CD4+ and CD8 + T cell immune response was analyzed by Cox proportional hazard models. Mean recipient age was 48 ± 13 years, with 73% male and 82% African American. Positive CMV specific CD4+ and CD8 + T cell immune response was found in 53% and 47% of the cohort at 1 month, 65% and 70% at 2 months, 80% and 75% at 4 months, 89% and 87% at 6 months, and 94% and 94% at 9 months post-transplant, respectively. There was no significant difference in the incidence of positive CMV specific T cell immune response between recipients of transplants from HCV aviremic donors compared to HCV viremic donors in unadjusted (for CD8+: HR = 1.169, 95%CI: 0.521–2.623; for CD4+: HR = 1.208, 95%CI: 0.543–2.689) and adjusted (for CD8+: HR = 1.072, 95%CI: 0.458–2.507; for CD4+: HR = 1.210, 95%CI: 0.526–2.784) Cox regression analyses. HCV viremia in donors was not associated with impaired development of CMV specific T cell immunity in this cohort.

## Introduction

Kidney transplants (KT) from Hepatitis C viremic/infected donors (HCV NAT+) to HCV negative recipients has become a major area of interest in the field of transplantation as an increasing number of centers are accepting and utilizing these kidneys over the last few years [1–3]. Excellent short-term and long-term patient and allograft survival have been undisputedly reported by various transplant centers across the United States (US) using two approaches – initiation of prophylactic Direct Acting Antiviral (DAA) drugs and a transmission-to-treat approach [4,5]. However, our previous work has shown that the incidence of first cytomegalovirus (CMV) infection was similar in HCV negative patients receiving the kidneys from HCV seropositive positive donors (HCV IgG Ab+) using the Scientific Registry of Transplant Recipients (SRTR) data set but might be higher than expected when transplanted from HCV viremic donors utilizing the transmission-to-treat approach, where evidence of HCV viremia in the recipients was required for the approval of DAA therapy from insurance agencies [3,6]. However, there is a paucity of data explaining the mechanisms of HCV and CMV interaction in acutely infected HCV transplant recipients.

CMV is a leading cause of morbidity and mortality in solid organ transplant recipients [7]. Understanding the mechanism of infection while protecting against CMV is crucial in immunocompromised KT recipients as CMV is associated with reduced patient and allograft survival, increased risk of rejection and coinfections [8–11]. Millions of dollars are spent yearly on CMV prophylaxis and treatment [12]. Higher incidence of CMV viremia in HCV negative transplant recipients of kidneys from HCV viremic donors can provisionally increase the health care cost burden, recurrent hospitalizations, and overall morbidity due to CMV related complications [13]. Recently, availability of new assays to detect CMV specific T cell immune response have shown promising results in preventing clinically significant CMV events by detecting recipient CD4+ and CD8 + T cell immunity against CMV [14,15]. Previous evidence has shown that asymptomatic CMV infections are associated with the presence of CMV specific CD4 + T cells prior to the emergence of CD8 + T cells in KT recipients and that CMV specific CD8 + T cells strongly correlate with protection against CMV disease [16,17]. However, there is a paucity of data showing the pattern of constitution of CMV specific CD4+ and CD8 + T cell immune response in KT recipients at various timepoints post-transplant. In addition, a majority of the US centers that transplant HCV infected kidneys into HCV negative recipients are using the prophylactic approach with standard 100-day CMV prophylaxis in low and intermediate risk groups and 200-day CMV prophylaxis for high-risk patients irrespective of tangible evidence of CMV specific CD4+ and CD8 + T cell immune responses [18].

Our study was designed to evaluate the role of donor HCV viremia in the development of CMV specific CD4+ and CD8 + T cell immune response at various timepoints post-transplant and to compare CMV specific CD4+ and CD8+ T cell immune response between groups of donors with differing HCV status at the same timepoints. We also sought to determine the overall proportions of KT recipients developing positive CMV specific CD4+ and CD8 + T cell immune responses at various timepoints post-transplant. We hypothesized that recipients receiving kidneys from HCV viremic donor would have reduced CD4+ and CD8 + T cell immune responses post-transplant compared to KT recipients from HCV aviremic donors (HCV NAT-).

## Materials and methods

### Cohort

**Figure 1.**
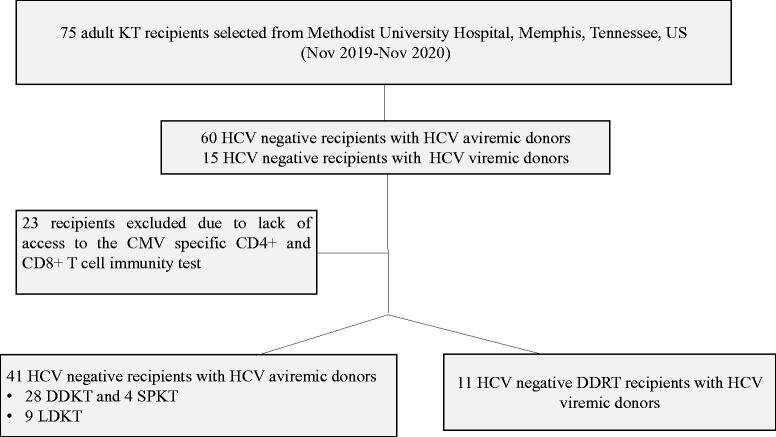
Flow chart of selection of the patients. *Abbreviations*: HCV; hepatitis C virus; KT: kidney transplant; DDKT: deceased donor kidney transplant; LDKT: living donor kidney transplant; SPKT: simultaneous pancreas kidney transplant; CMV: cytomegalovirus.

This was a prospective cohort study designed to enroll 75 adult HCV negative KT recipients from James D. Eason Transplant Institute, Methodist University Hospital (MUH), Memphis, Tennessee at the time of transplantation (Figure 1). Recruitment was started in November 2019 and everyone was followed up to total of one year. Sixty patients received KT from HCV aviremic donors whereas 15 received KT from HCV viremic donors. Twenty-three participants were never tested for CMV specific immune responses during the COVID pandemic when post-transplant care was switched to telemedicine and the test was not available at local laboratories closer to patients’ residences. The final cohort was composed of 52 patients with 41 receiving kidneys from HCV aviremic donors and 11 from HCV viremic donors. We included both deceased and living-donor KT recipients as well as simultaneous kidney-pancreas (SPK) transplant recipients. Recipient exclusion criteria were: age <18 years, pregnant and/or lactating females, simultaneous liver-kidney transplant, HCV negative recipients of kidneys from HCV antibody positive and nucleic acid amplification test (NAT) negative/aviremic donors, and recipients with no access to CMV specific CD4+ and CD8+ T cell response testing. Our center has a clinical protocol to measure CMV specific T cell response at specific timepoints after transplantation.

### Exposure

The primary exposure was KT from HCV viremic donor (NAT positive) versus HCV aviremic donor.

### Outcome

The primary outcome was development of CD4+ and CD8 + T cell immunity against CMV at 1, 2, 4, 6 and 9 months post-transplant by donor HCV status as measured by a commercially available intracellular flow cytometry assay (CMV inSIGHT™ T Cell Immunity Panel, Eurofins Viracor, Lee’s Summit, MO, USA using pp65 and/or IE-1 peptide pools as antigen). A positive test for immunity was defined as detection of ≥0.2% for both CD4 + T cells and CD8 + T cells with evidence of release of interferon-gamma (IFN-γ) on exposure to CMV antigen populations after subtracting the background.

The secondary outcome was development of CMV specific CD4+ and CD8 + T cell immune response at 1, 2, 4, 6 and 9 months post-transplant based on CMV risk stratification [7]. CMV risk stratification was assessed by the presence of immunoglobulin G (IgG) against CMV in recipients and donors. Low risk is defined as seronegative recipient and seronegative donor, intermediate risk as seropositive recipient and either seropositive or seronegative donor, and high risk as seronegative recipient and seropositive donor [19,20].

### Induction and maintenance immunosuppression and CMV prophylaxis

All patients received induction with rabbit anti-thymocyte globulin and were discharged on triple immunosuppression including calcineurin inhibitors, mycophenolate mofetil and prednisone. CMV high risk patient received total of 6 months, intermediate risk patients received total of 3 months valganciclovir treatment, while low risk patient received 3 months valaciclovir treatment.

### DAA protocol for HCV viremic transplants

HCV ribonucleic acid (RNA) viral load (VL) was measured in recipients *via* polymerase chain reaction in the first post-operative week or first outpatient clinic appointment and then weekly until becoming positive with reflex HCV genotype testing. Patients with detectable HCV RNA were started on a DAA regimen (glecaprevir/pibrentasvir, sofosbuvir/velpatasvir, or sofosbuvir/ledipasvir) for at least 12 weeks by MUH hepatologists. All DAA regimens were determined by hepatologist, and prescriptions were processed through a third-party payer [3,4].

### Monitoring

CMV specific T cell immune response was periodically checked at 1, 2, 4, 6, and 9 months posttransplant. Samples were collected during routine clinic visits at MUH according to the center’s clinical protocol and sent out to Eurofins Viracor Laboratory. Participants with occasional missing CMV specific T cell immune response testing due to coronavirus disease-2019 (COVID-19) pandemic related conditions were allowed to continue in the study. Once participants tested positive for CMV specific T cell immune response, they were always considered positive from that timepoint onwards for analytical purpose. If a participant had a missing test and a subsequent test result was negative for CMV specific T cell immune response, then the missing value was imputed as a negative result.

### Data management

The study was approved by the Institutional Review Board (IRB) (IRB# 20-07171-XP) by University of Tennessee Health Science Center. Informed consent was obtained individually from the recipients. The clinical and research activities being reported are consistent with the Principles of the Declaration of Istanbul as outlined in the ‘Declaration of Istanbul on Organ Trafficking and Transplant Tourism’. Data were extracted from the electronic medical record and the United Network for Organ Sharing (UNOS) system. All study data were collected, managed, and stored in the Research Electronic Data Capture (REDCap) tool hosted by the University of Tennessee Health Science Center [21].

### Statistical analysis

**Figure 2.**
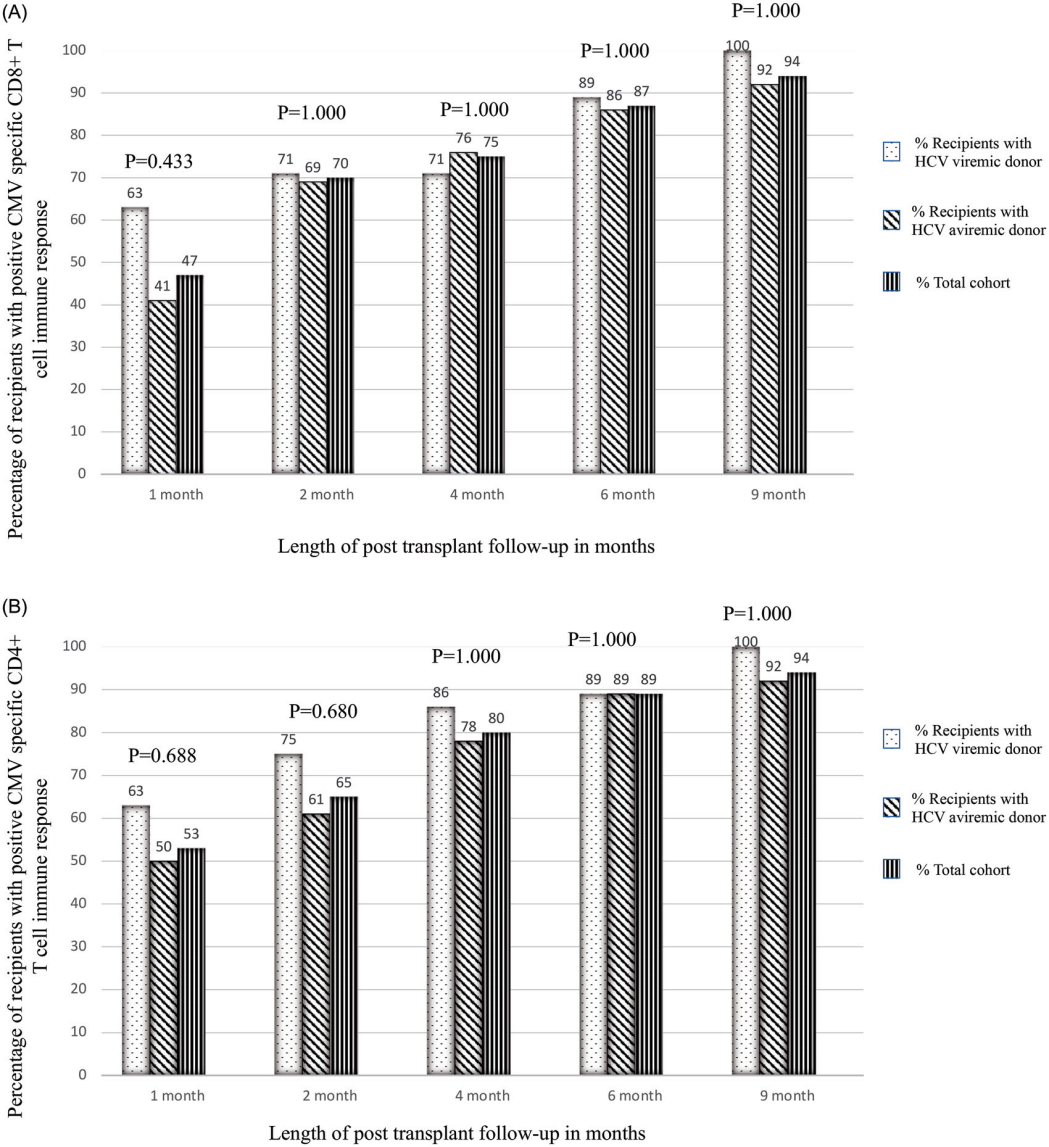
Proportion of recipients with positive CMV specific CD8+ (Panel A) and CD4+ (Panel B) T cell immune response in the cohort. *Abbreviations*: HCV: hepatitis C virus; CMV: cytomegalovirus.

**Figure 3.**
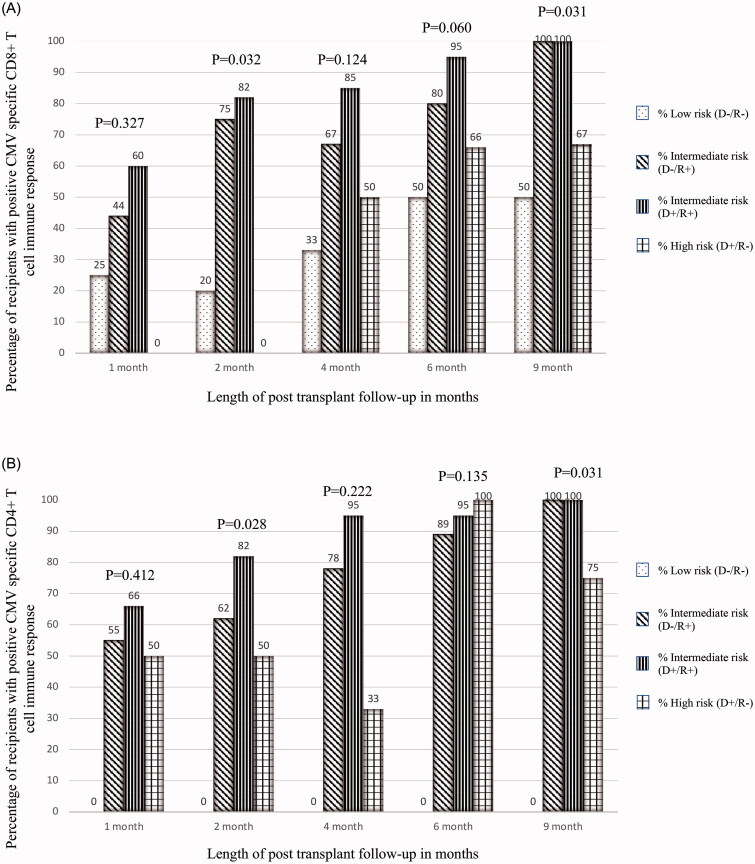
Proportion of recipients with positive CMV specific CD8+ (Panel A) and CD4+ (Panel B) T cell immune response in different CMV risk subgroups. *Abbreviations*: CMV: cytomegalovirus; D+: donor Ig G positive against CMV: D-: donor Ig G negative against CMV: R+: recipient Ig G positive against CMV: R-: recipient Ig G negative against CMV.

Baseline characteristics were determined for the entire cohort and individual groups based on HCV donor status. Data were presented as mean ± standard deviation (SD) or median and interquartile range (IQR) for continuous variables, and percent for categorical variables. Differences between groups were assessed by student *T*-test or Mann–Whitney test for continuous variables and chi-square-test (or Fisher’s exact test) for categorical variables. (Figures 2 and 3). Survival analysis was performed with the start of the observational period on the date of KT, and all recipients were followed up until the date of study completion or the censoring event of loss of follow-up without the development of the event of interest. The association between donor HCV status and recipient CMV specific CD4+ and CD8+ immune response was assessed using Cox proportional hazard models. We tested proportional hazards assumptions using scaled Schoenfeld residuals. The Cox regression analysis was adjusted for important biological covariates including gender, race, age and presence of diabetes mellitus in the recipients. We performed an additional survival analysis to test for associations between CMV risk subgroups and the incidence of CMV specific CD4+ and CD8 + T cell immune response. Reported p values were two-sided and defined as statistically significant if less than 0.05 for all analyses. All analyses were conducted using SAS Software ® Version 9.4.

## Results

### Baseline recipient, donor and transplant characteristics

Baseline characteristics of recipients and donors as well as transplantation related information categorized by donor HCV infection status are shown in Table 1. Recipients were predominantly male (73%), African American (82%) with Medicare as the primary insurance (80%). KT Recipients from HCV viremic donors were significantly older and tended to have higher body mass index (BMI).

**Table 1.** Baseline characteristics of the cohort.

Characteristics	Entire cohort (*n* = 52)	HCV aviremic (*n* = 41)	HCV viremic (*n* = 11)	*p*-Value
Recipient characteristics				
Age (years) – mean(SD)	48 (13)	46 (13)	55 (7)	**0.016**
Gender (male) – *n*(%)	38 (73)	29 (71)	9 (81)	0.705
BMI (kg/m^2^)-mean(SD)	29 (5)	28 (5)	31 (4)	**0.073**
Race – *n*(%)^a^				
Caucasian	7 (14)	6 (15)	1 (9)	
African American	42 (82)	32 (80)	10 (91)	1.000
Other	2 (4)	2 (5)	0 (0)	
Blood group – *n*(%)				
O	23 (44)	19 (47)	4 (36)	0.346
A	21 (40)	14 (34)	7 (64)	
B	5 (10)	5 (12)	0 (0)	
AB	3 (6)	3 (7)	0 (0)	
Marital status-*n*(%)				
Married	16 (30)	13 (32)	3 (28)	0.705
Single	26 (50)	22 (54)	4 (36)	
Other (divorced, separated or widowed)	10 (20)	6 (14)	4 (36)	
Insurance-*n*(%)				
Private	9 (20)	8 (24)	1 (10)	0.659
Medicare	34 (80)	25 (76)	9 (90)	
Dialysis vintage(months)median (IQR)	61 (34-110)	51 (28-104)	70 (58-122)	0.111
Cause of end stage kidney				
Disease-*n*(%)				
Hypertension	25 (48)	17 (42)	8 (73)	0.526
Diabetes	14 (27)	11 (27)	3 (27)	
Glomerulonephritis	6 (11)	6 (15)	0 (0)	
Cystic diseases	5 (10)	5 (12)	0 (0)	
Other inherited disease	1 (2)	1 (2)	0 (0)	
Unknown	1 (2)	1 (2)	0 (0)	
Comorbidity-*n*(%)				
Diabetes	24 (46)	19 (46)	5 (45)	1.000
Hypertension	49 (94)	39 (95)	10 (90)	0.517
PVD	1 (2)	1 (2)	0 (0)	1.000
CAD	10 (19)	6 (15)	4 (36)	0.189
COPD	5 (10)	2 (5)	3 (27)	**0.057**
Donor characteristics				
Type of donor-*n*(%)				0.176
Deceased donor	43 (83)	32 (78)	11 (100)	
Living donor	9 (17)	9 (22)	0 (0)	
Age (years) - median (IQR)^c^	30 (23-41)	27 (20-39)	32 (30-41)	0.172
Gender (male) - n(%)	36 (69)	25 (61)	11 (100)	**0.011**
Ethnicity-*n*(%)				1.000
Hispanic	1 (2)	1 (2)	0 (0)	
Non-Hispanic	51 (98)	40 (98)	11 (100)	
Race-*n*(%)^g^				**0.005**
Caucasian	31 (61)	20 (50)	11 (100)	
African American	18 (35)	18 (45)	0 (0)	
Asian	2 (4)	2 (5)	0 (0)	
BMI (kg/m^2^) – mean(SD)^i^	27 (6)	27 (7)	24 (4)	**0.062**
KDPI – median (IQR)^j^	42 (18-56)	33 (17-53)	49 (43-62)	**0.065**
DCD-*n*(%)	7 (13)	5 (12)	2 (18)	0.630
Cause of death-*n*(%)^f^				**0.007**
Anoxia	24 (57)	15 (48)	9 (82)	
Cerebrovascular/Stroke	1 (2)	1 (3)	0 (0)	
Head trauma	15 (36)	15 (48)	0 (0)	
Other	2 (5)	0 (0)	2 (18)	
IV drug use-*n*(%)	14 (32)	4 (12)	10 (100)	**<0.0001**
Blood Group-*n*(%)				0.424
O	23 (44)	19 (47)	4 (36)	
A	22 (42)	15 (36)	7 (64)	
B	5 (10)	5 (12)	0 (0)	
AB	2 (4)	2 (5)	0 (0)	
Comorbidity-*n*(%)^d^				
Diabetes	1 (2)	0 (0)	1 (10)	0.227
Hypertension	12 (27)	8 (23)	4 (40)	0.421
Smoker-*n*(%)^e^	13 (29)	9 (26)	4 (40)	0.448
HCV genotype-*n*(%)				
1a/1b	n/a	n/a	10 (91)	n/a
2a/2b	n/a	n/a	1 (9)	n/a
Transplant characteristics				
SPK-*n*(%)	4 (8)	4 (10)	0 (0)	0.566
Cross match-*n*(%)^b^				
HLA mismatches A				0.590
0	4 (8)	3 (7)	1 (9)	
1	20 (40)	17 (44)	3 (27)	
2	26 (52)	19 (49)	7 (64)	
HLA mismatches B				0.785
0	1 (2)	1 (3)	0 (0)	
1	17 (34)	14 (36)	3 (27)	
2	32 (64)	24 (61)	8 (73)	
HLA mismatches DR				**0.052**
0	6 (12)	5 (12)	1 (9)	
1	26 (52)	17 (44)	9 (82)	
2	18 (36)	17 (44)	1 (9)	
cPRA(%)-median (IQR)	7 (0-56)	14 (0-54)	3 (2-76)	0.469
Preformed DSA-*n*(%)^h^				
Absent	48 (100)	38 (100)	10 (100)	n/a
CMV status-*n*(%)				0.206
R-/D-	5 (9)	4 (10)	1 (9)	
R+/D-	14 (28)	8 (19)	6 (55)	
R-/D+	5 (9)	4 (10)	1 (9)	
R+/D+	25 (48)	22 (54)	3 (27)	
R+/D (unknown)	3 (6)	3 (7)	0 (0)	
EBV status-*n*(%)				0.397
R-/D-	7 (13)	7 (17)	0 (0)	
R+/D-	2 (4)	2 (5)	0 (0)	
R-/D+	41 (79)	30 (73)	11 (100)	
R+/D+	2 (4)	2 (5)	0 (0)	
rATG dose (mg)-mean(SD)	416 (95)	421 (102)	398 (61)	0.355
DGF-*n*(%)	7 (13)	7 (17)	0 (0)	0.322
Maintenance is at discharge-*n*(%)				
Prograf	17 (33)	11 (27) 30 (73)	6 (55)	0.144
Envarsus	35 (67)	38 (93)	5 (45)	0.144
Cellcept	49 (94)	3 (7)	11 (100)	1.000
Myfortic	3 (6)	41 (100)	0 (0)	1.000
Prednisone	52 (100)		11 (100)	n/a

*Abbreviations*: HCV: hepatitis C virus; BMI: body mass index; IQR: interquartile range; PVD: peripheral vascular disease; CAD: coronary artery disease; COPD: chronic obstructive pulmonary disease; HBV: hepatitis B virus; SPK: simultaneous pancreas kidney transplant; KDPI: kidney donor profile index; DCD: donation after cardiac death; PHS: public health services; IV: intravenous; HLA: human leukocyte antigen; cPRA: calculated panel reactive antibodies; DSA: donor specific antibodies; CMV: cytomegalovirus; EBV: Epstein Bar virus; rATG: rabbit anti-thymocyte globulin; DGF: delayed graft function; IS: immunosuppression.

Bold values highlighting where *p* < 0.1.

^a^Data for race was missing for total 1 patient (2%) belonging to the HCV negative donor group.

^b^Data for crossmatch was missing for total 2 patients (4%) and all belonging to the HCV negative donor group.

^c^Data for donor’s age was missing for total 1 patient (2%) belonging to the HCV negative donor group.

^d^Data for donor’s comorbid conditions was missing for total 8 patients (15%), seven belonging to the HCV negative donor group and one from HCV viremic donor group.

^e^Data for donor’s smoking status was missing for total 8 patients (15%), seven belonging to the HCV negative donor group and one from HCV viremic donor group.

^f^Data for donor’s cause of death was missing for total 10 patients (19%), all belonging to the HCV negative donor group.

^g^Data for donor’s race was missing for total one patient (2%) belonging to the HCV negative donor group.

^h^Data for preformed DSA was missing for total 4 patients (8%), 1 belonging to the HCV negative donor group and 3 from HCV viremic donor group.

^i^Data for donor’s BMI was missing for total 8 patients (15%) all belonging to the HCV negative donor group.

^j^Data for KDPI was missing for total 4 patients (8%) all belonging to the HCV negative donor group.

### Cohort immune response by donor HCV status

CMV Specific CD4+ and CD8 + T cell immune response was positive in 53% and 47% of the cohort at 1 month, 65% and 70% at 2 months, 80% and 75% at 4 months, 89% and 87% at 6 months, and 94% and 94% at 9 months post-transplant, respectively (Figure 2). via cox proportional hazards analysis, there was no significant difference in positive CMV specific T cell immune responses between KT recipients from HCV viremic donors versus HCV aviremic donors (for CD8+: HR = 1.072, 95%CI: 0.458–2.507; *p* = 0.561; for CD4+: HR = 1.210, 95%CI: 0.526–2.784; *p* = 0.654) adjusted for recipient age, gender, race and diabetes mellitus (Tables 2 and 3).

**Table 2.** Association between donor HCV status and recipient positive CMV specific CD8+ T cell immune response using Cox proportional hazard model for the entire follow-up period.

Variable	Hazard ratio	95% Confidence limits	*p* Value
Unadjusted model/Univariate			
Donor’s HCV status			
HCV aviremic	(Reference)	0.521–2.623	0.7042
HCV viremic	1.169		
Adjusted model/Multivariate			
Donor’s HCV status			
HCV aviremic	(Reference)		
HCV viremic	1.072	0.458−2.507	0.8734
Recipient’s age in years (+1 year)	1.016	0.988−1.046	0.2678
Gender			
Male	(Reference)		
Female	1.587	0.749–3.363	0.2277
Race			
Caucasian	(Reference)		
African American	2.132	0.638–7.124	0.2187
Diabetes Mellitus in recipient			
Absent	(Reference)		
Present	2.079	0.978−4.419	0.0570

*Abbreviations*: HCV: hepatitis C virus.

**Table 3.** Association between donor HCV status and recipient positive CMV specific CD4+ T cell immune response using Cox proportional hazard model for the entire follow-up period.

Variable	Hazard ratio	95% Confidence limits	*p* Value
Unadjusted model/Univariate			
Donor’s HCV status			
HCV aviremic	(Reference)		
HCV viremic	1.208	0.543–2.689	0.643
Adjusted model/ Multivariate			
Donor’s HCV status			
HCV aviremic	(Reference)		
HCV viremic	1.210	0.526–2.784	0.654
Recipient’s age in years (+1 year)	1.033	1.002–1.065	0.039
Recipient gender			
Male	(Reference)		
Female	2.587	1.087–6.158	0.032
Recipient race			
Caucasian	(Reference)		
African American	5.189	1.129–23.853	0.034
Recipient diabetes mellitus			
Absent	(Reference)		
Present	1.190	0.544–2.604	0.663

*Abbreviations*: HCV: hepatitis C virus.

### Cohort immune response by CMV risk status

The proportions of recipients with positive CMV specific CD4+ and CD8 + T cell immune response by CMV risk stratification throughout follow-up are shown in Figure 3 via adjusted Cox analysis and compared to CMV low risk patients, there was no statistically significant difference in CD8 + T cell immune response for CMV intermediate risk patients (for CD8+: adjusted HR =2.301, 95%CI: 0.272–19.483; *p* = 0.444) or CMV high risk patients (for CD8+: adjusted HR = 0.968, 95%CI: 0.083–11.233; *p* = 0.979). There was no event in the CD4+ T cell immune response low risk group, therefore we compared only CMV intermediate risk group with CMV high risk group for CD4+ T cell immune response via adjusted Cox analysis and compared to CMV intermediate risk patients, there was no statistically significant difference in CD4+ T cell immune response for CMV high risk patients (for CD4+: adjusted HR = 0.543, 95%CI: 0.117–2.515; *p* = 0.435).

## Discussion

We found no statistically significant difference in post-transplant CMV specific T cell immune response in HCV negative KT recipients from HCV viremic donors compared to HCV aviremic donors. The permissive HCV viremia that results from using a transmission-to-treat approach was thought to associate with higher incidence of CMV viremia in these recipients [3]. This phenomenon has been theoretically explained in prior literature in that HCV is known to have immunomodulatory properties that it uses for immune evasion to survive in the human host [22–24]. HCV modifies the T cell arm of the immune system, which is a major fighting arm against other viral infections like CMV [25,26]. Increased incidence of CMV coinfections have been reported in advanced HCV infected patients with liver fibrosis due to dysregulated Janus Kinase/Signal Transducer and Activator of Transcription (JAK-STAT) pathways adversely affecting the overall T cell response [27]. Other data in Human Immunodeficiency Virus (HIV) infected patients has shown that CMV IgG levels were higher in HCV/HIV co-infected women than in HIV mono-infected women, revealing an interaction between HCV viremia and CMV infection [28].

We found a non-significant trend toward higher proportion of recipients with HCV viremic donors developing CMV specific CD8 + T cell immunity at various timepoints post-transplant. The exact mechanism to explain this trend remains unknown however it can be argued that these findings indicate a possible role of HCV as an immune modifier with a positive effect on developing CD8 + T cell immunity against CMV, but statistical power was limited due to the small sample size.

We also determined the proportions of patients developing CMV specific T cell immunity at various timepoints post-transplant depending on their CMV risk stratification. Our results in this cohort of recipients with similar induction and maintenance immunosuppression corroborate the findings of previous work in the field showing that recipient CMV seropositivity is associated with early development of T cell immunity against CMV [29]. Our results further support the idea that CMV specific T cell immune response can be observed in CMV seronegative patients [30,31]. One possible explanation for this discrepancy is that cellular immunity in such cases is insufficient to induce humoral immunity that lasts for a longer time [30]. An unexpected finding was that 100% of the KT recipients from HCV viremic donors developed T cell immunity against CMV, but not until 9 months post-transplant. Of note, the KT recipients from HCV aviremic donors that never developed CMV specific CD8 + T cell immunity even at 9 months post-transplant were seronegative for CMV at the time of transplant. Our findings confirm the earlier observations of Thompson et al. that development of CMV specific T cell immunity post-transplant is a dynamic process, and recipients who do not initially have CMV specific T cell immunity can develop it later in the post-transplant course [29]. Reason(s) for undetectable CMV specific T cell immunity in some patients post-transplant remain(s) unclear. It is possible that some low risk patients were never exposed to CMV antigen resulting in no opportunity for developing CD8 + T cell immunity during the first months post-transplant; however, the absence of subsequent CMV specific CD8 + T cell immunity in high risk patients does not have a clear explanation other than the overall immunocompromised state.

A majority of transplant centers across the US, including our center, use fixed duration prophylactic valganciclovir dosing for CMV prevention, with its associated costs and risks, based on guidelines from the Transplantation Society International CMV Consensus Group [20,32–35]. Valganciclovir is known to cause resistant strains of CMV in some patients, and valganciclovir induced neutropenia can lead to a reduction in immunosuppression (typically mycophenolate or another antimetabolite), which can predispose to rejection [35,36].

Several studies have explored the utilization of CMV specific T cell immune response testing as a tool in predicting clinically significant CMV events in solid organ transplant recipients [37]. A previous randomized controlled clinical trial found that CMV specific T cell immune response guided discontinuation of prophylaxis for CMV disease in kidney transplant recipients resulted in significantly lower incidence of severe neutropenia but no significant increase in CMV replication or disease [38]. Based on our findings, a large proportion of patients are commenced on CMV prophylaxis despite specific T cell immunity against CMV very early in the transplant course. On the other hand, CMV prophylaxis in some high risk patients that may benefit from prolonged dosing is usually stopped at 6 months post-transplant based on current guidelines [20]. We believe our data can help individualize CMV prophylaxis strategies by providing deeper insight into the process for developing CMV specific T cell immunity post-transplant.

There are limitations to consider for our study. A major limitation is the small sample size, which was affected by the COVID-19 pandemic and its consequences. During this time, KT was deemed an elective surgery at our center, and KT volumes were significantly reduced, which also hampered study recruitment. Also, a substantial number of patients that consented to participate were not able to come to the hospital for the research blood draws, as follow-up visits were shifted to telemedicine and the test of interest was not available at their local laboratories. Moreover, we did not collect data CMV specific T cell immunity before transplantation. Another limitation is, we do not have data about CMV viremia and disease. However, out study goal was to assess developing CMV specific T cell immunity post-transplant.

Despite these limitations, the study provides an initial comparison of CMV specific CD4+ and CD8 + T cell immunity in KT recipients from HCV viremic donors versus HCV aviremic donors. To our knowledge, this is also the first report of the proportional trend of constitution of CMV specific CD4+ and CD8+ T cell immunity during the early post-transplant course in the KT population. We believe our single-center design with a standardized protocol for testing CMV specific T cell immunity and use of similar immunosuppression regimens between groups are significant strengths of our study. Additional questions raised by the study represent potential future areas for fruitful clinical research in the field of solid organ transplantation.

## Conclusion

In conclusion, recipients with KT from HCV viremic donors did not show significantly higher risk for CMV infection post KT based on CMV specific CD4+ and CD8 + T cell immune responses compared to recipients with KT from HCV aviremic donors. Our findings provide additional evidence that KT from HCV viremic donors to HCV negative recipients is a safe and valuable modality that does not impair the recipient’s ability to immunologically fight against CMV.

## Abbreviations

KT: kidney transplant
HCV: hepatitis C
CMV: cytomegalovirus
US: United States
DAA: direct-acting antiviral agent
HCV IgG Ab+: HCV seropositive positive donors
HCV NAT+: HCV viremic donors
HCV NAT-: HCV aviremic donors
JAK-STAT: Janus kinase/signal transducer and activator of transcription
HIV: human immunodeficiency virus
MUH: Methodist University Hospital
SPK: simultaneous kidney pancreas
IFN-γ: interferon-gamma
RNA: ribonucleic acid
VL: viral load
IRB: Institutional Review Board
BMI: body mass index
COPD: chronic obstructive pulmonary disease
EBV: Epstein Bar virus
DDKT: deceased donor kidney transplant
LDKT: living donor kidney transplant
SPKT: simultaneous pancreas kidney transplant
